# Nursing interventions to enhance self-care in heart failure: a systematic review interpreted through the COM-B model

**DOI:** 10.1186/s13643-026-03280-0

**Published:** 2026-08-12

**Authors:** Beti Kristinawati, Tintin Sukartini, Violeta Yuman Tanaya, Navian Fauzi Arifudin

**Affiliations:** 1 Doctoral of Nursing Student, Faculty of Nursing, Universitas Airlangga, Surabaya, Indonesia; 2 Department of Medical-Surgical Nursing, Nursing Study Program, Faculty of Health Sciences, Universitas Muhammadiyah Surakarta, Surakarta, Indonesia; 3 Department of Medical-Surgical, Emergency, Disaster, and Critical Care Nursing, Faculty of Nursing, Universitas Airlangga, Surabaya, Indonesia; 4 Nursing Study Program, Faculty of Health Sciences, Universitas Muhammadiyah Surakarta, Surakarta, Indonesia

**Keywords:** Heart failure, Self care, Nursing care, Health behaviour, Motivation, Randomised controlled trials

## Abstract

**Background:**

Heart failure (HF) is a long-term illness, frequently requiring hospital stay and having a significant morbidity and mortality rate. In order to avoid decompensation, lower readmission rates, and promote quality of life, adequate self-care is vital. This review synthesised nursing interventions intended to improve self-care among adults and older adults with HF and retrospectively interpreted their components using the Capability, Opportunity, Motivation–Behaviour (COM-B) model.

**Method:**

A systematic search was conducted on 11 November 2025 across seven electronic databases: PubMed, Scopus, Web of Science, CINAHL, Embase, MEDLINE, and PsycINFO. The review included randomised controlled studies evaluating behavioural, educational, psychological, or nursing interventions aimed at improving self-care among adults and older adults with HF. Risk of bias was assessed using the Joanna Briggs Institute Checklist for Randomised Controlled Trials. Database-specific search strategies and publication-year filters are reported in Additional file 1.

**Results:**

Nine studies met the inclusion criteria. The interventions included structured education, motivational interviewing, pre-discharge education, technology-based support, and multimodal programmes. Several studies reported improvements in self-care maintenance, management, or confidence; however, the direction and magnitude of effects varied across interventions and follow-up periods. Although none of the studies explicitly used the COM-B model, their intervention components could be retrospectively mapped to Capability, Opportunity, and Motivation.

**Conclusions:**

Nursing interventions may improve selected dimensions of HF self-care, particularly when education is combined with follow-up, motivational support, or practical monitoring resources. However, the evidence was heterogeneous, and the magnitude and consistency of effects remain uncertain. COM-B may provide a useful framework for interpreting intervention components, but the retrospective mapping does not demonstrate that COM-B mechanisms were prospectively tested.

**Systematic review registration:**

The procedure of this study was recently filed in the International Prospective Register of Systematic Reviews (PROSPERO) at the National Institute for Health Research (No: CRD420251237974).

**Supplementary Information:**

The online version contains supplementary material available at https://doi.org/10.1186/s13643-026-03280-0.

## Introduction

The primary cause of global morbidity, mortality, and the requirement for medical care is heart failure (HF), a complicated chronic cardiovascular disease [1–3]. HF globally affects around 64 million people, with roughly of 8–9 cases per 1000 individuals and a 20–25% 30-day rehospitalisation rate [4, 5]. This burden is increasing in Asia because of an ageing population and high cardiovascular risk factors, contributing to the growth in years lived with disability (YLDs) and disability-adjusted life years (DALYs) [6–8]. Progressive clinical circumstances of HF often cause a recurring decompensation cycle that diminishes functional ability, impacts quality of life, and raises the financial and psychosocial load on patients and families [9–11]. In long-term management, self-care skills play an essential role as they relate to symptom control, remission prevention, and improved life quality [12, 13].

Self-care is conceptualised in line with Riegel and colleagues’ theoretical work on chronic illness and heart failure self-care [14]. From this perspective, self-care refers to a process through which individuals maintain health through treatment adherence and lifestyle behaviours, monitor signs and symptoms, and respond appropriately when symptoms occur. In heart failure, this includes self-care maintenance, symptom monitoring/perception, self-care management, and self-care confidence [15]. The COM-B model was not used here to redefine self-care, but rather to interpret the behavioural determinants that may influence patients’ ability to perform self-care behaviours. Even though self-care has been acknowledged as a crucial part of managing heart failure, its application is still quite diverse due to individual, social, and environmental factors such health literacy, cognitive ability, caregiver support, and access to health resources [16, 17]. Various nursing interventions such as structured education, behavioural counselling, telemonitoring, and integrated disease management programmes have been developed to support self-care. Still, differences in intervention components, delivery methods, and implementation contexts make their effectiveness difficult to compare consistently [18, 19]. In addition, most interventions have not explicitly used the framework of health behavioural theory [20].

The COM-B model gives a more comprehensive theoretical viewpoint in explaining the drivers of self-care behaviour in HF patients [21]. The Capability, Opportunity, Motivation, Behaviour (COM-B) model provides a comprehensive behavioural framework for understanding the determinants of health-related behaviours [22]. According to this model, behaviour (B) occurs through the dynamic interaction of three key components: capability (C), opportunity (O), and motivation (M). Capability refers to an individual’s physical and psychological capacity to perform a behaviour, including knowledge and skills [23]. Opportunity encompasses external factors that enable or prompt the behaviour, such as environmental resources and social support. Motivation includes both reflective processes (e.g. beliefs, intentions, and goals) and automatic processes (e.g. emotions and habits) that influence behavioural engagement. Because self-care in heart failure involves complex behavioural processes, the COM-B framework offers a useful perspective for interpreting how different intervention components may support or hinder patients’ self-care behaviours. Unfortunately, no systematic review has been systematically synthesised nursing interventions and assessed the mechanisms of behaviour change through the COM-B framework. This gap limits the understanding of which intervention components are most effective in targeting patients' capabilities, opportunities, and motivation [24, 25].

This systematic review intends to identify and synthesise nursing treatments aiming at improving self-care in adults and older adults with HF. The findings are then interpreted through the COM-B model. By mapping the representation of skills, opportunities, and motives in diverse interventions, this study aims to provide an empirical and theoretical foundation for the creation of more effective and behaviour-change-oriented nurse interventions in long-term HF management.

## Methods

### Study design

This systematic review was reported in accordance with the Preferred Reporting Items for Systematic Reviews and Meta-Analyses (PRISMA) 2020 statement. The review protocol was prospectively registered in PROSPERO on 22 October 2025 (CRD420251237974).

### Search strategy

A systematic database search was conducted on 11 November 2025 in PubMed, Scopus, Web of Science, CINAHL, Embase, MEDLINE, and PsycINFO. These databases were selected to provide complementary coverage of biomedical, nursing and allied health, psychological and behavioural, and multidisciplinary literature relevant to HF self-care. Controlled vocabulary and free-text terms were combined for heart failure, adult and older populations, self-care, patient education, motivational interviewing, cognitive behavioural approaches, and other behavioural or psychosocial interventions.

The review considered peer-reviewed studies published from January 2015 to 11 November 2025. Search strategies were adapted to the indexing structure, controlled vocabulary, syntax, and filtering functions available in each database. Publication-year filters were applied where supported by the database platform. When a publication-year filter was unavailable or could not be incorporated into the search syntax, publication year was assessed manually during the study-screening process according to the predefined eligibility criteria.

All retrieved records were imported into EndNote for automated and manual duplicate removal and were subsequently managed in Rayyan during the screening process. No supplementary searches of reference lists, trial registries, or grey literature sources were undertaken. The complete database-specific search strategies, available filters, and numbers of records retrieved on 11 November 2025 are provided in Additional file 1.

### Eligibility criteria

Only studies matching the inclusion criteria were eligible:

Population: Hospitalised and non-hospitalised adults and older adults aged ≥ 18 years, clinically diagnosed with HF. No restrictions were applied for New York Heart Association (NYHA) class or comorbidity profile.

Intervention: Behavioural, educational, psychosocial, or lifestyle-focused interventions designed to improve HF self-care or its determinants. Eligible interventions included structured education, motivational interviewing, counselling, cognitive-behavioural approaches, discharge education, telephone or telehealth follow-ups, printed materials (e.g. manuals, booklets), and digital or technology-assisted tools. Interventions were required to report at least one measurable behavioural outcome (e.g. medication adherence, symptom monitoring, fluid or sodium restriction, daily weighing, exercise adherence, appointment keeping). Interventions were not required to explicitly reference the COM-B framework but needed to provide sufficient detail to enable mapping to Capability, Opportunity, and Motivation.

Comparator: Usual care or standard heart failure management, usual care generally included routine clinical management such as medication management, discharge education, and scheduled follow-up with healthcare providers.

Outcomes: Quantitative assessments of HF self-care behaviours or related behavioural characteristics measured using tested tools such as the Self-Care of Heart Failure Index (SCHFI) or the European Heart Failure Self-care Behaviour Scale (EHFScBS).

Study design: Peer-reviewed randomised controlled trials published in English from January 2015 to 11 November 2025, with full-text availability.

Exclusion criteria included pharmacological or device-only interventions without behavioural components; studies evaluating only knowledge, attitudes, or perceptions without behavioural outcomes; non-empirical publications (e.g. reviews, commentaries); The search was limited to studies published in English due to feasibility and resource considerations; paediatric or caregiver-only populations; and studies without full-text availability.

### Study selection

**Fig. 1:**
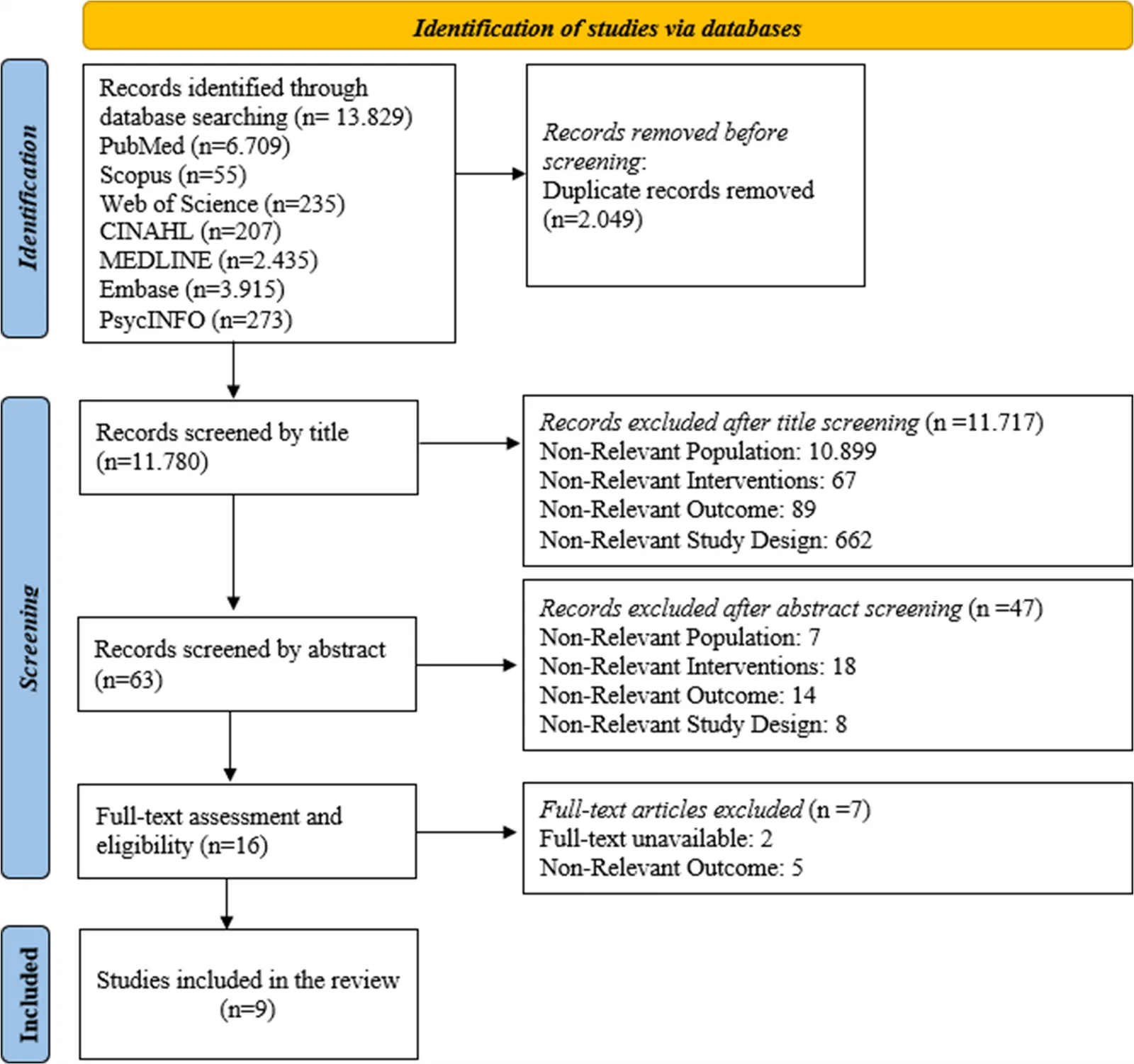
PRISMA flow diagram

Following duplicate removal in EndNote, two reviewers (B.K. and V.Y.T.) independently screened the titles of all retrieved records according to the predefined eligibility criteria. The abstracts of records retained after title screening were subsequently assessed. Potentially relevant articles were then evaluated in full text by B.K. and N.F.A. Reasons for exclusion during title, abstract, and full-text assessment were documented. Any disagreements were resolved through discussion or consultation with a third reviewer (T.S.) when required. The study-selection process is presented in the PRISMA flow diagram in Fig. 1.

### Data extraction

Data extraction was done through a standardised form that was pilot-tested on two studies by V.Y.T. and B.K. to ensure clarity as well as consistency in variable definitions and coding procedures. Following refinement, the form was applied to all included studies.

Two reviewers (V.Y.T. and B.K.) separately extracted information on the study's characteristics and participant demographics. The characteristics cover author, year, country, design, or setting while the demographics include sample size, age, or sex distribution. Moreover, clinical characteristics (NYHA class, comorbidities), intervention and comparator details, follow-up duration, and quantitative self-care outcomes were also extracted. The extracted variables were narratively summarised. Additional file 2 contains the full compiled features of all reviewed studies.

Intervention content was further mapped to the COM-B framework. Two reviewers (B.K. and V.Y.T.) independently assigned components to Capability (psychological or physical), Opportunity (social or physical), and Motivation (reflective or automatic). A third reviewer (T.S.) verified all extracted data and COM-B classifications for accuracy and completeness. The complete COM-B mapping matrix is provided in Additional file 4.

### Risk of bias assessment

To assess the methodological merit of the reviewed studies, The JBI Critical Appraisal Checklist for Randomized Controlled Trials was used Each study was graded as “yes”, “no”, or “unclear” across criteria relating to randomisation, concealed assignment, baseline comparability, blinding, completeness of follow-up, selective reporting, and overall trial design.

Risk-of-bias assessment was conducted independently by two reviewers (N.F.A and B.K). Any discrepancies between reviewers were discussed and resolved through in consultation with third reviewer as required (T.S.). This process ensured consistency and transparency in evaluating the methodological quality of the included trials. No study was excluded solely based on its risk-of-bias profile. Item-level assessments for each trial are available in Additional file 3.

### Synthesis approach

Marked heterogeneity was identified across studies in intervention content, methods of delivery, duration and frequency of follow-up, outcome measures, and reporting of effect estimates. These differences precluded a meaningful meta-analysis. A narrative synthesis was conducted to summarise intervention characteristics, reported effects, and patterns across studies. For each trial, the direction and statistical significance of changes in validated self-care outcomes were described, together with the intervention components mapped to the COM-B domains. In this review, self-care was considered the target behaviour of interest, in line with established theoretical frameworks of heart failure self-care.

The COM-B model was used as an analytic framework to interpret how intervention components may influence behavioural determinants of self-care. Because the included interventions were not originally designed using the COM-B framework, the model was applied retrospectively as a theory-informed interpretive approach. Intervention components described in each study were examined and mapped to the three COM-B domains: capability, opportunity, and motivation, based on their potential mechanisms in supporting patients’ engagement in heart failure self-care behaviours. The mapping process was conducted through iterative discussion among the review team to ensure conceptual consistency.

The narrative synthesis also considered the methodological quality of the included studies and the consistency of findings across trials. A formal GRADE assessment of the certainty of evidence was not conducted. Consequently, conclusions regarding the overall strength of the evidence were interpreted cautiously.

## Results

### Study selection

The database searches identified 13,829 records. After the removal of 2049 duplicate records, 11,780 records underwent title screening. Of these, 11,717 records were excluded because of non-relevant populations (*n* = 10,899), interventions (*n* = 67), outcomes (*n* = 89), or study designs (*n* = 662). The abstracts of the remaining 63 records were subsequently screened, resulting in the exclusion of 47 records because of non-relevant populations (*n* = 7), interventions (*n* = 18), outcomes (*n* = 14), or study designs (*n* = 8).

A total of 16 articles underwent full-text assessment. Seven articles were excluded because the full text was unavailable (*n* = 2) or because the study did not report a relevant self-care outcome (*n* = 5). Finally, nine randomised controlled trials were included in the qualitative synthesis. The study-selection process is presented in Fig. 1.

### Characteristics of included studies

The nine included randomised controlled trials comprised 1162 participants, with sample sizes ranging from 36 to 510 participants. Participants were generally adults and older adults with chronic heart failure, with mean ages spanning the late fifties to the early seventies. The proportion of female participants varied widely across studies.

Where reported, participants ranged from NYHA class II to IV, although most studies enrolled predominantly class II–III patients. Two studies included mainly class III patients, one study included mostly class III–IV patients, and two studies focused mainly on class II patients; however, NYHA class was not reported in all trials. Although the depth of clinical descriptions differed between studies, comorbidities including hypertension, diabetes mellitus, chronic obstructive lung disease, atrial fibrillation, and ischaemic heart disease were commonly noted.

Trials were conducted across diverse global regions, including Europe, the Middle East, East Asia, and Australia. While most interventions were initiated in hospital-based programmes, several incorporated post-discharge follow-up or home-based components. Follow-up durations ranged from less than three months to twelve months.

Self-care outcomes were measured using established instruments, most frequently the SCHFI and the EHFScBS. Symptom perception, self-care confidence, sleep quality, depression, and heart failure knowledge were among the other metrics. None of the included studies explicitly employed the framework of the COM-B model; nonetheless, all interventions incorporated behavioural components that facilitated subsequent mapping onto Capability, Opportunity, and Motivation. A thorough list of study characteristics is available in Additional file 2.

### Risk of bias

All nine trials were assessed using the JBI Critical Appraisal Checklist for Randomized Controlled Trials. No study was excluded on the basis of methodological quality. Most assessed domains were rated as having a low risk of bias. Blinding of participants and personnel was rated as unclear in two studies, whereas the remaining assessed domains were rated as low risk. Detailed study-level assessments are presented in Additional file 3.

### COM-B representation in interventions and self-care behaviours

#### Overall representation of COM-B

All intervention descriptions contained components that were retrospectively mapped onto Capability, Opportunity, and Motivation, although none of the studies explicitly applied the COM-B model. Interventions were delivered through a variety of formats, including structured education sessions, motivational interviewing, discharge teaching, telephone support, printed self-care materials, and technology-assisted strategies such as tablet-based monitoring platforms or avatar-based applications. Motivational interviewing was a prominent component in several interventions, while others relied on multimodal educational approaches or home-monitoring systems. The full COM-B mapping matrix is presented in Additional file 4.

#### Capability

Capability enhancements were primarily reflected in improved psychological capability, including disease-specific knowledge, symptom recognition, medication literacy, dietary management, and decision-making skills. Interventions that incorporated illustrated booklets, pre-discharge teaching, or nurse-led counselling sessions demonstrated consistent improvements in these areas.

Physical capability influenced patients’ ability to maintain daily self-care routines. Higher symptom burden, functional limitations, depression, or disrupted sleep were noted as barriers to self-care performance. Improvement in these symptoms was associated with increased self-care scores. Collectively, interventions, strengthening both cognitive understanding and practical skills produced notable gains in self-care maintenance and management.

#### Opportunity

Opportunity was manifested through the social and physical contexts that facilitated self-care. Social opportunity included caregiver involvement, sustained therapeutic relationships with nurses, and structured telephone follow-up. These components provided emotional support, reinforced daily routines, and enhanced continuity of care.

Physical opportunity was offered through environmental resources such as booklets, symptom diaries, home scales, and digital platforms for daily monitoring. Access to healthcare providers via hotline numbers or integrated care programmes further enabled timely action in response to worsening symptoms. Collectively, these factors supported the transition from capability to consistent behavioural performance.

#### Motivation

Motivation was reflected in both reflective and automatic processes. Reflective motivation was strengthened through motivational interviewing, goal setting, and the enhancement of self-efficacy. These strategies helped patients resolve ambivalence, clarify personal values, and increase readiness for behavioural change.

Automatic motivation emerged through habit formation supported by repeated monitoring cues, educational reminders, diaries, and digital applications. Regular reinforcement from nurses contributed to the establishment and maintenance of these habits. Interventions combining reflective motivational strategies with tools that supported habit building were particularly effective in sustaining behavioural change.

#### Adequacy of COM-B in explaining self-care determinants

Across all trials, the COM-B framework captured the key behavioural determinants associated with HF self-care. Capability explained cognitive and physical prerequisites. Opportunity captured social and environmental facilitators. Motivation, meanwhile, accounted for self-efficacy, internalisation, and habit formation.

However, contextual factors such as socioeconomic constraints, cultural influences, and household decision-making were rarely explored and may require additional frameworks for interpretation. In summary, COM-B provided a coherent and practical structure for interpreting behavioural mechanisms across the diverse interventions.

## Discussion

### Summary of key findings

This systematic review synthesised evidence from nine randomised controlled trials evaluating nursing interventions to foster self-care behaviours in adults and older adults with HF. This review provides a theory-informed perspective on randomised nursing interventions for heart failure self-care by interpreting intervention components through the COM-B behavioural model. Although none of the included studies explicitly used the COM-B model as their guiding framework, all interventions included components that could be interpreted within the COM-B domains. This suggests that the COM-B model may provide a relevant interpretive structure for understanding the behavioural components addressed by these interventions. Educational sessions, motivational interviewing, structured follow-up, technology-assisted monitoring, and caregiver involvement were among the strategies used to enhance self-care maintenance, management, and confidence [26]. Across several studies, reported improvements in self-care behaviours were associated with increased patient knowledge, supportive care environments, and strengthened motivation [27]. These findings are consistent with other evidence showing that self-care in chronic disease is influenced by a range of behavioural determinants.

### Capability as the foundation for behavioural change

The findings highlight capability, particularly psychological capability, an important component supporting self-care among patients with heart failure. Programmes that addressed disease-specific knowledge, symptom awareness, medication understanding, fluid and sodium management or decision-making skills reported improvements in self-care in several studies. This is consistent with earlier findings revealing associations among cognitive comprehension and health-literacy measures as well as treatment adherence in heart failure [15, 28]. Physical performance is also a determinant of intervention effects. Those with high symptom burden, functional limitations or comorbidities (including depression and sleep disorders) experience greater challenges in maintaining scheduled self-care. In a different setting, similar findings have shown that physical and psychological restrictions reduced adherence [29]. Notably, several studies reported that improvement in depressive symptoms was associated with enhanced self-care, underscoring the interplay between emotional capability and behavioural regulation [30].

Capability may be influenced by heart failure severity, particularly NYHA functional class. Patients with more advanced HF, such as those in NYHA class III or IV, often experience greater fatigue, dyspnoea, and reduced exercise tolerance, which may limit their physical capability to perform daily self-care activities, including symptom monitoring, physical activity, and adherence to dietary recommendations [31]. In contrast, patients with milder functional limitation, such as those in NYHA class I or II, may have greater physical reserve and therefore may be better able to engage in routine self-care behaviours. Disease severity may also affect psychological capability, as a higher symptom burden can complicate patients’ ability to interpret bodily changes and make timely self-care decisions.

Taken together, these results suggest that bolstering psychological and physical capability provides a cognitive and functional foundation upon which opportunity and motivation may act. Patient-facing education, repeated key messages, and simple instructional techniques may help strengthen patients’ capability to perform self-care.

### Opportunity as an external facilitator of self-care behaviour

Social and physical opportunities enable self-care. Self-care continuity is enabled by social factors, such as caregiver support, long-term nurse-patient relationship, and structured follow-up supports like phone calls and home visits. These opportunities reflect the protective factors social networks provide in chronic illness management [32]. Practical resources such as booklets, symptom diaries, monitoring tools, and telehealth services provide tangible opportunities [33]. These tools serve as reminders within the environment designed to prompt symptom tracking and decision-making [34]. System access to health professionals, specialised disease-management programmes, and communication enables immediate responsive actions to changing symptoms, consistent with the system-level facilitators described previously [35]. Overall, the findings suggest that Opportunity may help patients translate capability into self-care behaviour by providing resources and structured support.

Opportunity may also vary according to NYHA functional class. Patients with more severe functional impairment may require greater external support to sustain self-care behaviours, including caregiver assistance, closer nursing follow-up, and easier access to monitoring tools or structured discharge support [31]. In this context, social and environmental opportunities become particularly important in enabling self-care when patients’ physical condition limits independent action. Conversely, patients with less severe HF may rely less on intensive external support, although ongoing access to education and follow-up remains important for maintaining long-term self-care routines.

### Motivation as the primary driver of behavioural sustainability

Within the reviewed studies, both reflective and automatic motivation were represented in intervention components intended to support sustained self-care. Of the self-care and motivation studies, those utilising motivational interviewing techniques are the most successful at integrating reflective motivation as they resolve ambivalence as well as foster goal achievement and self-efficacy [36]. These findings are consistent with previous evidence suggesting that motivational interviewing may improve adherence and self-care confidence among individuals with heart failure [26, 37].

Motivation may likewise be shaped by disease severity. Patients with advanced HF may experience emotional distress, frustration, or reduced confidence related to persistent symptoms and functional decline, which may weaken motivation to maintain self-care behaviours over time [38]. At the same time, greater symptom burden may increase awareness of the seriousness of the condition and, in some patients, strengthen reflective motivation to adhere to treatment and monitoring recommendations. By contrast, patients with milder symptoms may have better confidence and routine engagement in self-care, but they may also perceive less urgency to sustain behavioural change [39]. These variations suggest that motivational components of nursing interventions may need to be tailored according to functional class and symptom burden.

The use of automated processes, driven by motivation, is designed to support habit formation such as maintaining weight logs, keeping symptom diaries, as well as responding to structured educational prompts. These help heart failure patients incorporate the self-care tasks into daily living [40]. Positive behavioural feedback loops, combined with regular nurse follow-ups, support behavioural science frameworks highlighting the role of habit cues and repeated reinforcement in sustaining long-term chronic disease self-care [41]. Overall, motivational components may contribute to the maintenance of behaviour, particularly when patients also have sufficient capability and opportunity to perform self-care.

### Strengths and limitations of the COM-B framework in the context of heart failure

The synthesis indicates that the COM-B framework offers a practical structure for interpreting self-care-related intervention components among heart failure patients. Capability, Opportunity, and Motivation were identifiable across the reported intervention components. The framework provided a useful structure for organising determinants such as knowledge, self-efficacy, social support, environmental resources, and motivational processes, consistent with established heart failure self-care theories [25, 42]. Yet, some limitations are apparent. Some contextual factors such as socioeconomic challenges, cultural patterns, and family decision-making processes are only superficially mentioned in the main studies. They are not completely incorporated into the COM-B without further reasoning. Other researchers have also pointed out that COM-B can also be further improved by integrating illness or culture-specific frameworks like the Middle-Range Theory of Self-Care of Chronic Illness [14, 43] or the Social Determinants of Health [44, 45]. Our interpretation of self-care was grounded in established heart failure self-care theory, particularly the work of Riegel and colleagues [15]. In this sense, COM-B should be understood as complementary rather than competing with self-care theory. The self-care framework clarifies the nature of the behaviours to be supported, whereas COM-B helps explain the behavioural conditions under which those behaviours are more likely to occur [23]. This combined perspective strengthened our interpretation of how educational, supportive, and motivational nursing interventions may improve heart failure self-care.

As none of the interventions are designed and implemented using COM-B, the review depends on a deductive mapping of the components of the interventions. This limits the usefulness of the model as a structured design tool. Nevertheless, the reported intervention components were broadly consistent with COM-B domain, suggesting that the model may be relevant as an interpretive framework in heart failure behavioural research.

Overall, the body of evidence suggests that nursing-related interventions may improve multiple dimensions of HF self-care, particularly when they combine education with follow-up or motivational support. However, the certainty of this evidence should be interpreted cautiously because of heterogeneity in intervention content, comparator conditions, follow-up duration, and outcome measures.

### Limitations of the review

This review has several limitations. First, the included studies varied considerably in sample size, intervention content, comparator conditions, follow-up duration, outcome measures, and analytical approaches. This heterogeneity limited direct comparisons across studies and precluded a meaningful meta-analysis. Most self-care outcomes were measured using self-report instruments, which may have introduced recall and social desirability bias. In addition, socioeconomic and cultural factors were incompletely reported in the primary studies, limiting the interpretation of how these contextual factors may have influenced self-care behaviours and intervention outcomes.

Second, the content of usual care varied across studies, ranging from minimal written information to routine education and follow-up support. These differences may have contributed to variations in the observed intervention effects. The review was also restricted to English-language publications, which may have introduced language bias. Trial registries, grey literature sources, and the reference lists of included studies were not searched, and the full texts of two potentially relevant articles were unavailable. Consequently, some relevant published or unpublished evidence may have been missed.

Finally, the COM-B model was applied retrospectively because none of the included interventions was originally designed using this framework. Although the mapping process was conducted systematically, the classification of intervention components relied on reviewer judgement and may not fully reflect the mechanisms intended by the original investigators. Furthermore, the certainty of the evidence was not formally assessed using GRADE. Therefore, the findings regarding intervention effectiveness and the interpretation of COM-B components should be considered cautiously.

### Implications for nursing practice

This systematic review highlights the importance of psychological capability for nurses’ ability to provide nursing care. This can be achieved by fostering nurse competency via patient-centred educational experiences, providing clear instructional resources, and supporting skill development through practical experience. Nurses need to assess their patients’ emotional or cognitive barriers such as depression, anxiety, or low levels of health literacy. Moreover, they need to address these barriers by providing appropriate support.

Caregivers should support patients’ self-care by facilitating communication, using therapeutic communication techniques, and providing follow-up through structured phone calls or home monitoring programmes. Symptom diaries, digital monitoring devices, and other devices can help reinforce the daily self-care routines.

It is important to note that the interventions included in this review were not originally developed using the COM-B framework. Instead, the COM-B model was applied retrospectively as an interpretive lens to examine how different intervention components may influence behavioural determinants of self-care. Therefore, the mapping presented in this review should be interpreted as a theory-informed synthesis rather than a direct test of COM-B mechanisms. Nevertheless, the framework offers a useful structure for understanding how educational, supportive, and motivational elements of nursing interventions may interact to facilitate self-care behaviours in patients with heart failure. Strategies for promoting motivation, such as motivational interviewing and ongoing positive reinforcement, should be incorporated into everyday nursing practice to increase self-efficacy and help sustain healthy behaviours. The COM-B model provides a holistic framework for nurses in developing individualised care plans to address all three of the behavioural determinants, including providing care that is culturally appropriate.

## Conclusions

This systematic review identified educational, motivational, follow-up, and technology-assisted nursing interventions intended to support self-care among adults and older adults with HF. Several studies reported improvements in selected self-care domains; however, the findings varied across interventions, outcome measures, and follow-up periods.

Retrospective mapping indicated that intervention components could be interpreted within the Capability, Opportunity, and Motivation domains. However, none of the included studies prospectively tested COM-B mechanisms, and the mapping should therefore be understood as a theory-informed interpretation rather than evidence of causal mechanisms. COM-B may provide a useful structure for developing and reporting future nursing interventions, but further theory-based studies with longer follow-up are needed.

## Supplementary Information

Additional file 1: Full database search strategies (DOCX). *Description:* Complete search strings (syntax) for all databases (PubMed, Scopus, Web of Science, CINAHL, EMBASE, MedLine, PsycInfo).

Additional file 2: Detailed study characteristics table (DOCX). *Description:* Detailed characteristics of all included randomized controlled trials, including design, setting, country, sample size, age, sex distribution, NYHA class, comorbidities, intervention and comparator details, follow-up duration and self-care outcome measures.

Additional file 3: JBI risk-of-bias assessment table (DOCX). *Description:* Item-level Joanna Briggs Institute Critical Appraisal Checklist ratings for each included randomized controlled trial.

Additional file 4: COM-B mapping matrix (DOCX). *Description:* Detailed mapping of intervention components in each trial onto Capability (psychological/physical), Opportunity (social/physical) and Motivation (reflective/automatic) domains of the COM-B model.

## Data Availability

This published article and its supplementary materials include all data generated or analysed during this systematic review. Additional file 1 contains the full database search strategies (syntax), Additional file 2 contains the reviewed study characteristics table, Additional file 3 contains the JBI risk-of-bias assessment table, and Additional file 4 contains the COM-B mapping matrix.
