# Supplementary material for: Nursing interventions to enhance self-care in heart failure: a systematic review interpreted through the COM-B model
Source: Syst Rev. 2026 Aug 12;15:239. doi: 10.1186/s13643-026-03280-0 (PMC13540883; doi:10.1186/s13643-026-03280-0)
Supplement: Supplementary file 1 [file 13643_2026_3280_MOESM1_ESM.docx]

**Full Database Search Strategies**

This appendix presents the full search strategies used in each database and the number of records retrieved on 11 November 2025. Publication-year filters were applied where supported by the database platform. For databases in which a publication-year filter was unavailable or could not be incorporated into the search syntax, publication year was verified manually during screening according to the predefined eligibility criteria.

| **Database** | **Query** | **Result** |
| --- | --- | --- |
| PubMed | ((((((((((((((Heart Failure[MeSH Terms]) OR ("Cardiac Failure"[Text Word])) OR ("Congestive Heart Failure"[Text Word])) OR (HF[Text Word])) OR (CHF[Text Word])) AND (Adult[MeSH Terms])) OR (Adult*[Text Word])) OR (Aged[MeSH Terms])) OR (Elder*[Text Word])) OR (Geriatr*[Text Word])) OR ("Older adult*"[Text Word])) OR (Middle Aged[MeSH Terms])) OR ("Middle Age"[Text Word]) AND (y_10[Filter])) AND (((((((((((((((((((((((((Patient Education as Topic[MeSH Terms]) OR ("Patient Education"[Text Word])) OR ("Education of Patients"[Text Word])) OR (Health Education[MeSH Terms])) OR (Health Literacy[MeSH Terms])) OR (Motivational Interviewing[MeSH Terms])) OR (Cognitive Behavioral Therapy[MeSH Terms])) OR ("Cognitive Behavioral Therap*"[Text Word])) OR ("Cognition Therap*"[Text Word])) OR (Behavior Therapy[MeSH Terms])) OR ("Behavior Therap*"[Text Word])) OR ("Conditioning Therap*"[Text Word])) OR ("Behavioral Support"[Text Word])) OR ("Behavioral Techniq*"[Text Word])) OR ("Behavior Treatment*"[Text Word])) OR (Patient Participation[MeSH Terms])) OR ("Patient Involvement"[Text Word])) OR ("Patient Engagement"[Text Word])) OR ("Patient Activation"[Text Word])) OR ("Patient Empowerment"[Text Word])) OR (Psychotherapy[MeSH Terms])) OR (Psychotherap*[Text Word])) OR (Psychosocial Intervention[MeSH Terms])) OR ("Psycho* Intervention*"[Text Word])) OR (Psychoeducation*[Text Word]) AND (y_10[Filter]))) AND ((((((((Self Care[MeSH Terms]) OR ("Self-Care"[Text Word])) OR (Self-Management[MeSH Terms])) OR ("Self Management"[Text Word])) OR (Self Efficacy[MeSH Terms])) OR (Health Behavior[MeSH Terms])) OR ("Health Behavior*"[Text Word])) OR ("Health Related Behavior*"[Text Word]) AND (y_10[Filter])) | 6.709 |
| Scopus | ( TITLE-ABS-KEY ( "Heart Failure" OR "Cardiac Failure" OR "Congestive Heart Failure" OR HF OR CHF ) AND TITLE-ABS-KEY ( Adult* OR Aged OR Elder* OR Geriatr* OR "Older adult*" OR "Middle Aged" OR "Middle Age" ) AND TITLE-ABS-KEY ( "Patient Education as Topic" OR "Patient Education" OR "Education of Patients" OR "Health Education" "Health Literacy" OR "Motivational Interviewing" OR "Cognitive Behavioral Therapy" OR "Cognitive Behavioral Therap*" OR "Cognition Therap*" OR "Behavior Therapy" OR "Behavior Therap*" OR "Conditioning Therap*" OR "Behavioral Support" OR "Behavioral Techniq*" OR "Behavior Treatment*" OR "Patient Participation" OR "Patient Involvement" OR "Patient Engagement" OR "Patient Activation" OR "Patient Empowerment" OR Psychotherapy OR Psychotherap* OR "Psychosocial Intervention" OR "Psycho* Intervention*" OR Psychoeducation* ) AND TITLE-ABS-KEY ( "Self Care" OR "Self-Care" OR "Self-Management" OR "Self Management" OR "Self Efficacy" OR "Health Behavior" OR "Health Behavior*" OR "Health Related Behavior*" ) ) AND PUBYEAR > 2015 AND PUBYEAR < 2026 | 55 |
| WOS | "Heart Failure" OR "Cardiac Failure" OR "Congestive Heart Failure" OR HF OR CHF (Topic) and Adult* OR Aged OR Elder* OR Geriatr* OR "Older adult*" OR "Middle Aged" OR "Middle Age" (Topic) and "Patient Education as Topic" OR "Patient Education" OR "Education of Patients" OR "Health Education" "Health Literacy" OR "Motivational Interviewing" OR "Cognitive Behavioral Therapy" OR "Cognitive Behavioral Therap*" OR "Cognition Therap*" OR "Behavior Therapy" OR "Behavior Therap*" OR "Conditioning Therap*" OR "Behavioral Support" OR "Behavioral Techniq*" OR "Behavior Treatment*" OR "Patient Participation" OR "Patient Involvement" OR "Patient Engagement" OR "Patient Activation" OR "Patient Empowerment" OR Psychotherapy OR Psychotherap* OR "Psychosocial Intervention" OR "Psycho* Intervention*" OR Psychoeducation* (Topic) and "Self Care" OR "Self-Care" OR "Self-Management" OR "Self Management" OR "Self Efficacy" OR "Health Behavior" OR "Health Behavior*" OR "Health Related Behavior*" (Topic) and 2025 or 2024 or 2023 or 2022 or 2021 or 2020 or 2018 or 2019 or 2017 or 2016 (Publication Years) | 235 |
| CINAHL | TX ("Heart Failure" OR "Cardiac Failure" OR "Congestive Heart Failure" OR HF OR CHF) AND TX (Adult* OR Aged OR Elder* OR Geriatr* OR "Older adult*" OR "Middle Aged" OR "Middle Age") AND TX ("Patient Education as Topic" OR "Patient Education" OR "Education of Patients" OR "Health Education" "Health Literacy" OR "Motivational Interviewing" OR "Cognitive Behavioral Therapy" OR "Cognitive Behavioral Therap*" OR "Cognition Therap*" OR "Behavior Therapy" OR "Behavior Therap*" OR "Conditioning Therap*" OR "Behavioral Support" OR "Behavioral Techniq*" OR "Behavior Treatment*" OR "Patient Participation" OR "Patient Involvement" OR "Patient Engagement" OR "Patient Activation" OR "Patient Empowerment" OR Psychotherapy OR Psychotherap* OR "Psychosocial Intervention" OR "Psycho* Intervention*" OR Psychoeducation*) AND TX ("Self Care" OR "Self-Care" OR "Self-Management" OR "Self Management" OR "Self Efficacy" OR "Health Behavior" OR "Health Behavior*" OR "Health Related Behavior*") | 207 |
| MEDLINE | (((((Heart Failure or (heart failure or cardiac failure) or Congestive heart failure) and Adult) or (adult or adults) or Aged or (aged or elderly) or Middle Aged or (middle aged or middle age)).mp.) and ("Patient Education as Topic" or ("education of patients" or patient education) or Health Education).mp. or Health Literacy/ or Motivational Interviewing.mp. or Cognitive Behavioral Therapy.mp. or (cognitive behavioral therapy or cognition therapy or cognition therapies or cognitive behavior therapy).mp. or Behavior Therapy.mp. or (behavior therapy or behavior therapies or behavior treatment or behavioral support or behavioral techniques).mp. or Patient Participation.mp. or (patient involvement or patient engagement or patient activation or patient empowerment).mp. or Psychotherapy.mp. or Psychosocial Intervention.mp. or Psychoeducation.mp.) and (Self Care or (self care or self-care) or Self-Management or (self-management or self management) or Self Efficacy or Health Behavior or (health behavior or health behaviors or health related behavior)).mp.) | 2.435 |
| Embase | (((((heart failure or (cardiac failure or chronic heart failure) or Congestive heart failure) and adult) or adults or aged or elderly or middle aged or middle age).mp.) and (patient education or (education, patient or "patient education as topic") or health education or health literacy or motivational interviewing or cognitive behavioral therapy or (cognitive behavior therapy or cognitive behavior treatment or cognitive behavioral treatment or cognitive behaviour therapy or cognitive behaviour treatment or cognitive behavioural therapy or cognitive behavioural treatment)).mp. or behavior therapy/ or (behavior treatment or behavioral therapy or behaviour therapy or behaviour treatment).mp. or patient participation.mp. or patient involvement.mp. or psychotherapy.mp. or psychosocial intervention.mp. or psycho-social intervention.mp. or psychoeducation.mp.) and (self care or (self management or self-management or selfcare) or self efficacy or health behavior or (health behaviour or health related behavior or health related behaviour)).mp.) | 3.915 |
| PyscINFO | (((((Heart failure or Congestive heart failure or Chronic heart failure) and Adult) or (Middle Aged or middle aged adult) or Geriatrics).mp.) and (Client Education or "educate the patient" or Health Education or Health Literacy or Motivational Interviewing or Cognitive Behavior Therapy or Cognitive Therapy or Behavior Therapy or (Behavior Therapy or Cognitive Therapy) or Client Participation or Patient Participation or Psychotherapy or Psychosocial Interventions or Psychoeducation).mp.) and Self-Care or Self-Management or Self Management or Self-Efficacy or Self Efficacy or Health Behavior or (Health behavior or Health Related Behavior)).mp.) | 273 |
