# Supplementary material for: Nursing interventions to enhance self-care in heart failure: a systematic review interpreted through the COM-B model
Source: Syst Rev. 2026 Aug 12;15:239. doi: 10.1186/s13643-026-03280-0 (PMC13540883; doi:10.1186/s13643-026-03280-0)
Supplement: Supplementary file 2 [file 13643_2026_3280_MOESM2_ESM.docx]

**Table 1** General Characteristics of Reviewed Studies

| **Study ID** | **Design / setting** | **Country** | **Sample size (n) / Female (%)** | **Mean age, years (SD)** | **Clinical profile (dominant NYHA; main comorbidities)** | **Intervention vs control** | **Follow-up** | **Self-care outcome measure(s)** |
| --- | --- | --- | --- | --- | --- | --- | --- | --- |
| Navidian et al. (2017) (41) | RCT, inpatients | Iran | 82 (41 vs 41); 52.4% female | 58.3 ± 11.8 | NYHA not reported; comorbidities not reported | 4 in-hospital motivational interviewing (MI) sessions over last 4 days of admission focusing on HF self-care vs conventional individual education with self-care booklet | 8 weeks post-discharge | SCHFI v6.2 (self-care maintenance, management, confidence) |
| Carlson et al. (2019) (42) | RCT, post-discharge | Not reported | 84 (45 vs 39); 47.6% female | 72.6 ± 12.9 | NYHA not reported; common comorbidities: hypertension, arthritis, diabetes, COPD, cancer, renal disease, asthma, prior MI, stroke | Healthy Heart Tracker booklet with situational guidance “what to do when symptoms occur” vs usual HF education materials (“red folder”) | 2- and 6-weeks post-discharge (<3 months) | SCHFI v7.1 (maintenance, symptom perception, management, confidence) |
| Hägglund et al. (2015) (43) | RCT, home-based | Sweden | 72 (32 vs 40); 31.9% female | 75 ± 8 | Mostly NYHA III; frequent comorbidities: diabetes, COPD, hypertension, atrial fibrillation, prior MI, renal disease | Home-based Heart failure Information System (tablet linked to scale, daily monitoring and self-management tips) vs written HF information sheet and priority phone number | 90 days (≈3 months) | EHFScB-9; HF-specific HRQoL (KCCQ); generic HRQoL (SF-36); HF knowledge (Dutch Heart Failure Knowledge Scale) |
| Chen et al. (2020) (44) | RCT, inpatients and follow-up | Taiwan | 62 (28 vs 34); 37.1% female | 57.4 ± 13.3 | Predominantly NYHA II–III; comorbidity burden moderate (Charlson index ≈3) | Structured, tailored HF education with illustrated booklet during admission plus 5 post-discharge telephone follow-ups vs routine predischarge HF education and single discharge information sheet | 1, 3, 6, 12 months (12-month duration; main effects within 6 months) | SCHFI subscales (maintenance, management); sleep (PSQI); depression (PHQ-9) |
| Chen et al. (2018) (45) | RCT, inpatients | China | 62 (29 vs 33); 27.4% female | 59.8 ± 7.1 | Mostly NYHA III–IV; comorbidities not reported | 4 in-hospital nurse-led MI sessions plus telephone follow-ups at weeks 2, 4, 8 vs conventional HF education during admission plus routine follow-up calls | 2 months post-discharge (<3 months) | SCHFI (maintenance, management, confidence) |
| Al-Sutari el al. (2017) (46) | RCT, mixed setting | Jordan | 144 (72 vs 72); 59.7% female | 64.7 ± 9.9 | Predominantly NYHA III; comorbidities not reported | Multimodal HF education: 1 individual session, simple self-care manual, plus structured telephone follow-up for 3 months vs usual cardiology follow-up without structured education or calls | 3 months | SCHFI (maintenance, management) |
| Iovino et al. (2021) (47) | RCT, outpatients | Italy | 510 (340 vs 170); 42% female | 74 (median) | Mostly NYHA II; main comorbidity: ischemic HF | Three arms: MI for patients only; MI for patients plus caregiver; standard care (medical follow-up). All received written HF education | <3 months | SCHFI v6.2 (maintenance, management) |
| Wonggom et al. (2020) (48) | RCT, pragmatic | Australia | 36 (17 vs 19); 19.4% female | 67.5 ± 11.3 | Predominantly NYHA I–II; comorbidities: prior MI, peripheral vascular disease, cerebrovascular disease, chronic lung disease | Avatar-based HF educational application at home plus usual care vs usual HF education and booklet only | 30 and 90 days (<3 months) | SCHFI v6.2 (maintenance, management, confidence) |
| Köberich et al. (2015) (49) | RCT, hospital-based | Germany | 110 (58 vs 52); 17.3% female | 61.7 ± 12.0 | Predominantly NYHA II; comorbidities: dilated cardiomyopathy, ischemic heart disease, valvular disease, hypertrophic cardiomyopathy | Standardised individual HF self-care education session (~66 minutes) plus booklet, diary and 4 follow-up calls over 3 months vs usual medical care without nurse HF education | 3 months | German 9-item EHFScBS; HF-specific QoL (KCCQ); care dependency |
