# Supplementary material for: Nursing interventions to enhance self-care in heart failure: a systematic review interpreted through the COM-B model
Source: Syst Rev. 2026 Aug 12;15:239. doi: 10.1186/s13643-026-03280-0 (PMC13540883; doi:10.1186/s13643-026-03280-0)
Supplement: Supplementary file 3 [file 13643_2026_3280_MOESM3_ESM.docx]

**Table 2** Risk of Bias Assessment (JBI)

| **Study ID** | **Random sequence generation** | **Allocation concealment** | **Blinding of participants and personnel** | **Blinding of outcome assessment** | **Incomplete outcome data** | **Selective outcome reporting** | **Other bias** |
| --- | --- | --- | --- | --- | --- | --- | --- |
| Navidian et al. (2017) (41) | Low | Low | Unclear | Low | Low | Low | Low |
| Carlson et al. (2019) (42) | Low | Low | Low | Low | Low | Low | Low |
| Hägglund et al. (2015) (43) | Low | Low | Low | Low | Low | Low | Low |
| Chen et al. (2020) (44) | Low | Low | Low | Low | Low | Low | Low |
| Chen et al. (2018) (45) | Low | Low | Low | Low | Low | Low | Low |
| Al-Sutari el al. (2017) (46) | Low | Low | Low | Low | Low | Low | Low |
| Iovino et al. (2021) (47) | Low | Low | Unclear | Low | Low | Low | Low |
| Wonggom et al. (2020) (48) | Low | Low | Low | Low | Low | Low | Low |
| Köberich et al. (2015) (49) | Low | Low | Low | Low | Low | Low | Low |
