# Supplementary material for: Nursing interventions to enhance self-care in heart failure: a systematic review interpreted through the COM-B model
Source: Syst Rev. 2026 Aug 12;15:239. doi: 10.1186/s13643-026-03280-0 (PMC13540883; doi:10.1186/s13643-026-03280-0)
Supplement: Supplementary file 4 [file 13643_2026_3280_MOESM4_ESM.docx]

**Table 3.** Mapping of COM-B Components Across Included Studies

| **Study** | **Capability** | **Opportunity** | **Motivation** |
| --- | --- | --- | --- |
| Navidian et al. (2017) (41) | Knowledge of heart failure, recognition of symptom, correction of misconceptions, ability to link symptoms to appropriate actions | Counselor support through in-hospital motivational interviewing sessions | Increased intrinsic motivation, reduced ambivalence, enhanced self-efficacy and commitment to change |
| Carlson et al. (2019) (42) | Health literacy, comprehension of written instructions, ability to recognize and respond to symptoms | Living conditions, availability of booklets, access to written educational materials | Perceived health status, preference for educational materials, motivation to adhere to self-care recommendations |
| Hägglund et al. (2015) (43) | Knowledge of heart failure, ability to use tablet devices, understanding of medication regimens and symptom monitoring | Home-based tablet, connected weighing scale, clinic contact number, technical support | Sense of control, confidence in disease management, development of daily monitoring routines |
| Chen et al. (2020) (44) | Knowledge and decision-making skills, understanding of self-care, reduction in depression and sleep disturbances | Family support, access to telephone consultations, relationship with educators | Self-efficacy, desire to prevent readmission, motivation to maintain healthy behaviours |
| Chen et al. (2018) (45) | Correction of incorrect knowledge about symptoms and management, improved understanding of care plans | One-to-one therapeutic relationship with nurses, ongoing telephone support | Intrinsic motivation, realistic goal setting, increased self-efficacy and confidence in symptom management |
| Al-Sutari el al. (2017) (46) | Knowledge of heart failure, recognition of worsening symptoms, understanding of diet, fluid restriction and medication | Family involvement in education, regular telephone contact | Self-care confidence, regimen adherence, internalisation of the importance of self-care behaviours |
| Iovino et al. (2021) (47) | Understanding of self-care principles, ability to share tasks with caregivers | Caregiver support, family engagement during motivational interviewing sessions, access to healthcare services | Self-efficacy, optimism, reduced ambivalence toward change, increased readiness to engage actively in self-care |
| Wonggom et al. (2020) (48) | Health literacy, understanding educational content delivered via avatar, problem-solving ability related to symptoms and management | Nurse support, home-accessible avatar-based educational application, availability of booklet | Self-efficacy, social persuasion via avatar, enhanced confidence in performing self-care behaviours |
| Köberich et al. (2015) (49) | Knowledge of heart failure, ability to monitor symptoms and weight, increased self-efficacy | Nurse support, booklet, symptom-monitoring diary, level of independence in daily activities | Confidence in self-care, motivation to reduce dependency on others for symptom management |
